# The effect of short-time microwave exposures on *Listeria monocytogenes* inoculated onto chicken meat portions

**Published:** 2015-06-15

**Authors:** Tayebeh Zeinali, Abdollah Jamshidi, Saeid Khanzadi, Mohammad Azizzadeh

**Affiliations:** 1 *Department of Food Hygiene and Aquaculture, Faculty of Veterinary Medicine, Ferdowsi University of Mashhad, Mashhad, Iran; *; 2 *Department of Clinical Sciences, Faculty of Veterinary Medicine, Ferdowsi University of Mashhad, Mashhad, Iran.*

**Keywords:** Chicken meat, *Listeria monocytogenes*, Microwave

## Abstract

*Listeria monocytogenes* can be found throughout the environment and in many foods. It is associated primarily with meat and animal products. *Listeria monocytogenes* has become increasingly important as a food-borne pathogen. The aim of this study was to evaluate the effect of microwave (MW) treatment of chicken meat samples which were inoculated with *L. monocytogenes*. Drumettes of broiler carcasses were soaked in fully growth of *L. monocytogenes* in Brain-Heart Infusion broth. The swab samples were taken from the inoculated samples, after various times of radiation (10, 20, 30, 40, 50, 60, 70 and 80 sec), using a domestic MW oven at full power. Following exposures, viable counts and surface temperature measurements were performed. The bacterial counts were performed on Oxford agar. The results indicated that equal or longer than 60 sec exposures of chicken portions to MW heating which enhances the median surface temperature more than 74 ˚C could eliminate the superficial contamination of chicken meat with *L. monocytogenes. *Statistical analysis showed samples with equal or longer than 60 sec exposures to MW heating had significant decrease in population of inoculated bacteria compared with positive control group (*p *< 0.05). Pearson correlation showed a significant correlation between the bacterial population and temperature of samples due to MW exposure (*p *< 0.001, *r *= – 0.879 and *r*^2^ = 0.773).

## Introduction

In the genus of *Listeria*, which cause the infection of Listeriosis in both animals and human, *Listeria mono-cytogenes* is a major pathogenic microorganism.^1^ Listeriosis caused by *L. monocytogenes* has been increased drastically in the recent years.^2^ The microorganism is present in soil, water, vegetables, and intestinal contents of a variety of birds, fish, insects and other animals.^3^ Various dairy products, meat products and types of seafood have been reported to be contaminated with this pathogen and are implicated in sporadic as well as epidemic cases of listeriosis.^4^^,^^5^

In human, the illness may range from mild flu-like sickness to severe manifestations. It is associated with septicemia, meningoencephalitis and abortion, primarily affecting pregnant, new-born, and immunocompromised individuals.^2^ Death is rare in healthy adults, however, can occur at a rate as high as 30% in persons at highest risk.^6^ Because of its ability to survive and proliferate at refrigeration temperature^7^ and due to its ubiquitous character, *L. monocytogenes *easily enters the human food chain and may multiply rapidly.^8^

The standard in some countries, require absence of *L. monocytogenes* in 25 g of foods (zero tolerance) while other countries have a tolerance of below 100 CFU per g of *L. monocytogenes* at the point of consumption. Finally, some countries, have a tolerance of below 100 per g for some foods and a zero tolerance for others, especially those which are supportive of growth and with extended shelf-lives.^9^

Several decontamination methods on food surfaces have been introduced, including thermal and non thermal treatments. Microwave (MW) can be classified in physical non-thermal treatment. 

High frequency energy including MW and radio-frequency energy belongs to the non-ionizing radiations. Microwaves lie between the infrared and radio frequency portions of the electromagnetic spectrum.^10^ In a MW oven the heating of food results from molecular friction between water molecules under an oscillating electric field of specific frequency.^11^ Heating by MW energy is used for several purposes, e.g., cooking, pasteurization, sterilization and blanching of foods.^12^^,^^13^ The safety of MW cooking in relation to foodborne pathogens is questioned. There are studies reporting complete inactivation of microorganisms including pathogens, in inoculated cooked foods or reheated in MW ovens.^13^^,^^14^ The aim of the present study was to investigate the effect of different times of MW heating on the fate of* L. monocytogenes*, inoculated onto drumette of broiler carcasses.

## Materials and Methods

**Equipment and samples.** Microwave irradiation was performed in a MW oven (Model MF45; Samsung, Seoul, Korea), with a rotating glass plate, a frequency of 2,450 MHz, and power of 900 watts. The MW was used at full power for heating the chicken portions. A Number of 27 fresh drumette of broiler carcasses, sold in wrapped packages, obtained from a local market. All samples were transferred to the laboratory within 1 to 2 hr at 4 ˚C in insulated boxes and stored at 4 ˚C until use within 24 hr after purchase. The drumette of broiler carcasses was treated with H_2_O_2_ + Ag^+^ (Sanosil; Kimiafam, Tehran, Iran) according to manufacturer's instruction at room temperature as a sanitizer and washed three times with sterile distilled water to remove the residuals. All samples were examined for any pre-existing contamination with* L. monocytogenes* following the method described by Vanderzant and Splittstoesse.^15^

**Preparation of the** ***L. monocytogenes*** **inocula.** Pure cultures of* L. monocytogenes* were prepared by sub culturing the test strain (*L. monocytogenes*: ATCC 7644) into 500 mL of brain heart infusion broth (BHI; Merck KGaA, Darmstadt, Germany), following incubation at 32 ˚C for 24 hr. The concentration of the resulting culture was determined by preparing serial dilutions and viable count by surface plating on Oxford *Listeria* selective agar (Merck KGaA, Darmstadt, Germany). This culture media were used for inoculation of the chicken meat samples.

The absorbance of the cultured media were also determined in 600 nm wave length, using a spectrophotometer (Model 6105; Jenway, Essex, UK), to inoculate the same dose of bacteria in repeating the experiment. 

**Inoculation** **procedure** **and** **microbiological** **analysis. **The 27 portions of chicken drumettes were immersed into 500 mL of the prepared *L. monocytogenes* suspension for 10 min. They were drained by dripping on absorbent sterile cheesecloth for another 10 min and they were placed in sterile glass Petri dishes. Three samples were reserved for estimating *L. *monocytogenes concentration of the tested portions. The remaining 24 samples were divided into eight equal groups and each group was heated into the MW oven operating at full power for 10, 20, 30, 40, 50, 60, 70 and 80 sec, respectively. Surface temperatures in the approximate center of the upper surface of the samples were measured immediately after exposures, by a thermometer placed beneath the skin of the irradiated sample.

 Following MW heating, after about ten minutes using a template the upper surfaces of the samples were swabbed (wet and dry swabbing method).^15^

To determine the numbers of surviving *L. monocytogenes* after each exposure, decimal dilutions from each swabs containing tube were prepared and total viable count were performed by surface plating on the Oxford *Listeria* selective agar following incubation at 32 ˚C for 24 hr. 

**Statistical analysis. **The statistical analysis was performed using SPSS statistical software (Version 16; SPSS Inc., Chicago, USA). A non-parametric Kruskal-Wallis test at *p* < 0.05 was used to determine the effect of time of MW exposure on *L. monocytogenes* viability. Pairwise comparison of viability of *L. monocytogenes* between positive control and other groups were investigated by the Mann-Whitney U test considering Bonferroni adjustment. The relationship of inoculated bacterial population viability with temperature of samples due to MW exposure was examined with Pearson correlation test.

## Results

Pre-existing contamination with *L. monocytogenes* was not detected in any chicken meat samples. The concentration of cultured media inoculated with *L. monocytogenes* was determined as 1.6 10^6^ CFU mL^-1^, using total viable count method,^15^ and its absorbance at 600 nm determined equal as 0.640.

**Fig. 1:**
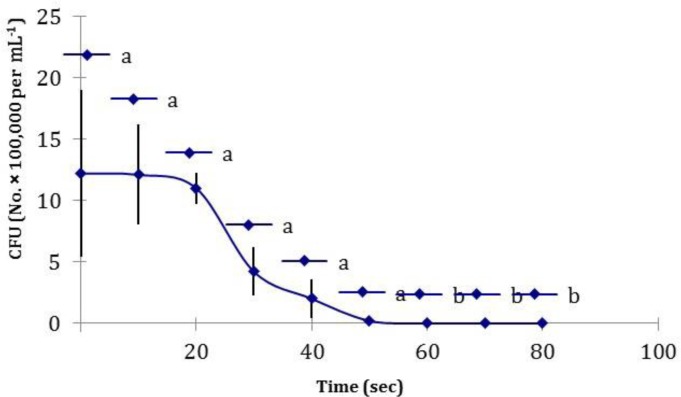
Destruction of *Listeria monocytogenes* as a function of microwave exposure time in drumettes of broiler carcasses. Values followed by different superscript letters are significantly different (*p* < 0.05).

**Fig. 2:**
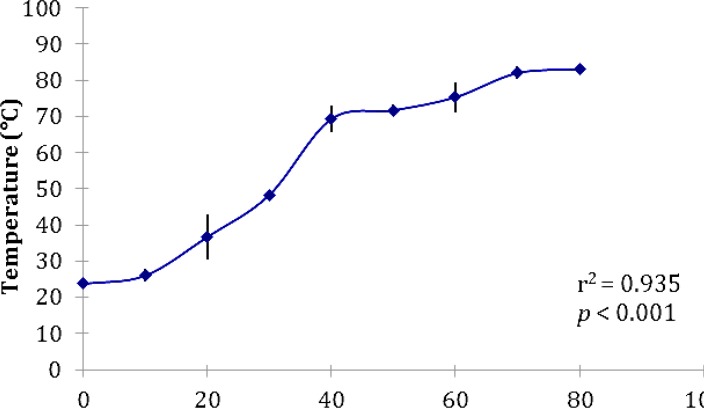
Final surface temperature of drumettes after different time of microwave exposure.

The bactericidal kinetics of MW for *L. monocytogenes* and for different exposure times is shown in Figure 1 and Final surface temperature of drumettes after different time of microwave exposure is shown in Figure 2. 

Elimination of *L. monocytogenes* was observed after the end of 60 sec exposure time, when the median surface temperature was increased to 74 ˚C.

Statistical analysis showed samples with equal or longer than 60 sec exposures of chicken portions to MW heating had significant decrease in population of inoculated bacteria compared with positive control group (*p* < 0.05). Less than 60 sec of MW exposure didnot have significant difference in regard to population of inoculated bacteria. Pearson correlation showed a significant correlation between the bacterial population and temperature of samples due to MW exposure (*p *< 0.001, *r *= – 0.879 and *r*^2^ = 0.773).

## Discussion

Microwave ovens have become common household appliances in developed countries and, to some extent, in developing countries. This relatively inexpensive technology is commonly used to cook or warm foods in homes, offices, and some restaurants. With respect to consumer safety, the research reported here showed that MW radiation could be used to control (to reduce or sometimes to completely eliminate) microbial potential pathogens in food. 

Evidence suggests that MWs are being used more frequently than ever before to cook raw foods. Although MW reheating has been shown to be a generally reliable method of reducing microbiological pathogens, little research has been performed on its efficacy to promote microbiological safety in cooking raw foods.^16^

According to our study induction of 74 ˚C superficial temperature in chicken meat portions could eliminate the inoculated bacteria, which its primary contamination rate with* L. monocytogenes* was 1.6 10^6^ CFU mL^-1^. The duration of radiation with full power to produce this temperature was 60 sec. 

A 5-log reduction of the viable count was also reported for *E. coli* suspension exposed to full power of MW radiation (600 watts) in 80 sec.^17^ In another study MW radiation which produced an internal temperature of 85 ˚C in fresh whole roasting chickens, could eliminate *Salmonella typhimurium*.^18^ Also, in a study by Jamshidi*et al*.,^19^ the MW radiation which enhance the surface temperature more than 70 ˚C, could eliminate the superficial contamination of cattle beef slices with *E. coli *O157:H7. Although the inoculated bacteria in our study were eliminated after 60 sec of MW exposure, however, it should be noted that other parameters like size and shape of the radiated meat samples may influence the elimination of inoculated bacteria, because in an study on chicken breast portions, elimination of *E. coli* O157:H7 cells occurred after 35s of MW exposure at 73.7 ˚C, however, when whole chickens were exposed to MW radiation, even with 92 ˚C in some area, viable cells of *E. coli* O157:H7 were recovered from all samples of whole chicken.^20^ In another study survival rate of pathogens such as *L. monocytogenes* and *Salmonella* *spp*. in food heated in MW ovens, is attributed to the non-uniform heating and their asymmetrical form.^16^^,^^18^

Extreme variability of surface and subsurface temperatures has been reported by other researchers in meat samples heated by MWs, however, the central area is where the least temperature increase is expected to occur.^16^^,^^21^ In our study only surface temperature measurements were taken from the sample’s center. Measuring the temperature of sample’s centre also prevents the ''edge-heating effect'' which is overheating of corners and edges of foods in a MW field, caused primarily by the uneven energy distribution during MW heating.^22^

In our study the swabbing of samples were performed after ten minutes of MW exposure, because it was reported that post-heating holding times of two or more minutes, increases bacterial destruction.^14^ Survival of some inoculated pathogens in meat portions after MW exposure may be due to immediate sampling. The primary concern associated with MW cooking is uneven heat distribution, which results in the formation of hot and cold spots in the food.^16^ To guarantee microbiological safety it has been recommended to cover the food with wax paper and checking temperature in at least three different sites.^16^

In conclusion, consumers can use MW ovens to significantly reduce microbial pathogens in foods like chicken meat portions. Microwave irradiation is a cost-effective, practical, fast, easy, and safe method of decontaminating foods.
